# Perceptions of medical students on narrow learning objectives and structured debriefing in medical escape rooms: a qualitative study

**DOI:** 10.1186/s12909-024-05295-4

**Published:** 2024-04-11

**Authors:** Tami Jørgensen, Oscar Rosenkrantz, Kristine Elisabeth Eberhard, Theo Walther Jensen, Peter Dieckmann

**Affiliations:** 1 Copenhagen Academy for Medical Education and Simulation (CAMES), Capital Region of Denmark, Borgmester Ib Juuls Vej 1, 2730 Copenhagen, Denmark; 2 Department of Cardiology, Bispebjerg Hospital, Copenhagen, Denmark; 3 Department of Anaesthesia, Center of Head and Orthopaedics, Copenhagen University Hospital - Rigshospitalet, Copenhagen, Denmark; 4 Copenhagen University Hospital - Rigshospitalet, Copenhagen, Denmark; 5 Prehospital Center, Region Zealand, Denmark; 6 Department of Public Health, University of Copenhagen, Copenhagen , Denmark; 7 Department of Quality and Health Technology, University of Stavanger, Stavanger, Norway

**Keywords:** Gamification, Innovative teaching, Escape room, Debriefing, Learning objectives

## Abstract

**Background:**

Escape rooms are increasingly used in medical education as a complementary learning technique or even alternative to traditional educational approaches. Few studies focus on debriefing following medical escape rooms and how escape rooms can be used to achieve pre-defined learning objectives. Evaluating the use of narrow learning objectives may increase the depth of reflections and transform an engaging team event into an effective learning opportunity. This study aimed to explore participants’ experiences and perceived learning outcomes of narrow learning objectives in a medical escape room with debriefing.

**Methods:**

In this explorative, qualitative study, participants saw a video lecture, participated in an escape room experience, and in a following debriefing. Throughout this learning session, the learning objectives concerned “exchange of information” and are therefore relatively narrow. Participants then participated in a semi-structured focus group interview and completed a demographic questionnaire. Participants were volunteer final-year medical students. Focus group interview recordings were transcribed and analysed using systematic text condensation.

**Results:**

Thirty-two students in eight groups completed the study. Five themes were described in the analysis of the focus group interviews: Experience with the narrow learning objectives, topics discussed in the debriefing, learning mechanisms, learning outcomes concerning exchange of information and influences of the learning approach.

**Conclusions:**

Narrow learning objectives and structured debriefing seem to increase perceived learning depth of medical escape room sessions. Using semi-structured debriefing still allows for discussions of other elements relevant to the students.

**Clinical trials:**

Clinical.trials ID NCT04783259.

**Supplementary Information:**

The online version contains supplementary material available at 10.1186/s12909-024-05295-4.

## Background

There is increasing evidence that medical students prefer interactive education styles with elements of gamification [1–3]. Gamification is applying game mechanics to a non-gaming environment [4], which improves achievement of learning goals compared to traditional teaching methods [5–7].

One interactive gamification technique is escape rooms, a themed exercise that involves solving puzzles and riddles to get out of a room within a specific time limit [8]. An example of an educational escape room is the Medical Escape Room Game Experience (MERGE) [9]. It is designed to raise awareness about non-technical skills (NTS) [10, 11] among healthcare students by presenting medically themed logic- and skill-based puzzles to be solved as a team. NTS are defined as the social, cognitive and personal management skills necessary for safe and effective performance. These skills are important across various high risk industries including nuclear power, aviation and healthcare [12].

Like simulations, escape rooms are experiential learning settings. Compared to a simulation, however, participants engage less in role-play and more in a game. Where a simulation at least sometimes asks participants to assume a professional role other than their own, participants in an escape room typically enter as “themselves.”

In such a learning situation, participants share the experience but perceive it from different angles. Debriefing can enlighten differences and strengthen the learning outcome from experiential learning situations by allowing reflection on the educational experience [13–15]. Therefore, debriefing will supplement an escape room’s inherent entertainment value to increase learning [16]. Further, the debriefer can be a peer to the learners as peer-to-peer feedback is suggested to affect the learning outcome positively [17].

The considerable number of debriefing structures published indicate that there is value in organising the debriefing in one way or another. The research group also experienced that structure in the debriefing is appreciated by participants and facilitators. On a theoretical basis, structured debriefings might positively affect the collaboration between facilitators and participants, as both know what to expect, once the structure is established [18, 19].

In a debriefing, learning objectives can be predetermined [14, 20] with narrow or broad wording. The research group differentiates between narrow and broad learning objectives. Narrow learning objectives concern focused and well-defined questions as opposed to broad learning objectives that are more open and likely to spur many different discussions depending on the learner. The “breadth” metaphor is always relative: “Knowing how errors occur” or “Discussing communication” are examples of broader learning goals with many possible subtopics whereas “Understanding the role of eye contact in non-verbal communication” in contrast is relatively narrow. When using narrow rather than broad learning objectives, the discussion can reach deeper reflection levels, as fewer topics are covered [21, 22]. The discussion might not cover topics of interest to participants if they are outside the narrow learning objectives, resulting in discussions being terminated during a debriefing.

Only four studies evaluated escape room debriefing [23–26] and concluded that participants would have preferred more structured debriefing relating to specific outcomes for the escape room sessions.

Published studies applied broad learning objectives or had no pre-set learning objective. Thus, no knowledge exists about how narrow learning objectives in a medical escape room are perceived by participants and how they affect the learning experience. The research group believes this knowledge might optimise the overall learning outcome of medical escape rooms by helping educators choose suitable learning objectives. Focusing on learning objectives during debriefing can optimise learning and emphasise the educational character of these entertaining activities. When having learning objectives tailored to the needs of the participants, it is, in the research group’s experience, easier for the educator to provide a high-quality learning session. This can be done by emphasising certain aspects of the learning objectives (e.g., spending more time on discussing them) to satisfy the learning needs and wishes of the participants.

For other experiential learning settings, like simulation, debriefing was declared the “heart and soul” of learning [27]. Therefore, the research group assumes that debriefing is valuable for escape rooms as well. Given the richness and openness of the learning situation in an escape room it is unclear whether the debriefing should focus on “everything” or specific potentials in the situation. Both approaches likely have advantages and disadvantages.

This study aimed to explore participants’ experiences and perceived learning outcomes of narrow learning objectives in a medical escape room with debriefing.

## Methods

This was a qualitative study using semi-structured focus group interviews and text condensing. The research group was interested in exploring participants’ perceptions and needed a method that allowed participants to express those experiences. Given the character of the learning objectives, the cognitive aspects of participants’ learning were of interest. Therefore, verbal descriptions in an interview would be a valuable method to collect data and answer the research question [28]. The research group operated within the constructivist paradigm as it tried to understand a phenomenon from the perspective of those experiencing it.

This section describes the approach, but the supplementary material should be read to understand the experimental work clearly.

### Setting

The escape room followed the MERGE manual [9] and was conducted at Copenhagen Academy of Medical Education and Simulation (CAMES) at Herlev Hospital, Denmark. The theme was a zombie apocalypse. It consisted of seven medically themed, logic- and skill-based puzzles that had to be solved sequentially, and the award at the end was the cure for the fictive zombie virus. The MERGE ‘Triage’ puzzle was exchanged with a puzzle box with laparoscopic forceps, focussing on teamwork (see Appendix 2). Behind a see-through mirror, a facilitator monitored the escape room events. Participants had 45 min to solve the puzzles. If they struggled in progress, the facilitator provided planned scenario lifesavers to help keep the time frame [29]. All the faculty had experience facilitating experiential learning settings, including simulation and escape room experiences.

### Data collection

Following the escape room, participants were interviewed semi-structured in focus groups and the individuals involved answered a questionnaire about their experience, perceived learning outcome, and demographic information (see Appendix 1).

The puzzles in the escape room were in English, while participants communicated in Danish. The video lecture, debriefing, focus group interview, and questionnaire were in Danish. Illustrative citations from the condensation process were translated from Danish into English.

### Participants

Participants were medical students who had completed four out of six years of their studies at the University of Copenhagen (UCPH), Denmark. Participants had completed at least four months of internship, experienced clinical practice close to that experienced by young physicians, and had some experience with simulation. Participants were recruited via social media, signed up in groups of four to five, and chosen based on the order of application. Participants did not receive any compensation.

### Intervention

The intervention was a structured learning session comprising four parts: a video lecture, focused instructions before the escape room, the escape room scenario, and a post-session debriefing. It was conducted in March 2021.

The learning session focused on two narrow learning objectives: “*Recognising the different ways of exchanging information*” and “*Discussing the impact of exchanging information on problem solving*”. These were chosen based on previous focus points and learning wishes by former participants [9]. The first learning objective concerned knowledge and comprehension of Bloom’s taxonomy, and the second concerned application and analysis [30].

The video lecture concerned theory of exchange of information in general terms, thus preparing participants to work with the concrete learning objectives and was developed within the research team (see Appendix 3). The focused instructions included practical information on the escape room’s course and emphasised the need to focus on exchange of information, as it was the learning objective. Debriefing was a semi-structured conversation steered by TJ, who has practical experience in the peer-to-peer debriefing of medical students and facilitated the discussion following a manual (Appendix 4) based on an established debriefing model [13].

### Semi-structured focus group interviews

Immediately after debriefing, participants were focus group interviewed with a semi-structured interview guide by KE or PD (see interview guide, Appendix 5). Focus group interviews concerned participants’ experiences and perceived learning outcomes of narrow learning objectives in a medical escape room with debriefing. Some of the main questions explored how they felt about the format, if and why participants would have preferred a less structured format and whether or not they felt limited by the narrow focus of the debriefing. Furthermore, participants were asked when they experienced learning outcomes and what these were.

Focus group interviews were estimated to last 30 to 45 min and were video and audio recorded. Interviewers emphasised that all points of view were relevant and essential, including perceived challenges.

### Analysis

Focus group interviews were transcribed ad verbatim by TJ and OR and analysed using systematic text condensation [31]. Condensation focussed on participants’ statements. Unclear and explicitly irrelevant citations (e.g. chit-chat) were excluded. The coding was done in Microsoft Excel. The citations were loaded into one column, where each row represented a different speaker. After initially reading the focus group interview transcripts several times, the coding proceeded with paraphrasing each cell in the next column on a similar level of abstraction by TJ. Themes were assigned to each paraphrase, condensing content of the focus group interviews. Themes were used by TJ and OR to identify all citations relevant to the study aim. These steps were repeated until researchers concluded that saturation had been reached by watching the remaining focus group interviews, and no more codes or themes were identified. TJ condensed the statements, selecting and translating representative citations from Danish to English before grouping them into main themes. Three research group members not involved in the coding and condensation (KE, TWJ, PD) cross-checked the coding and condensation process.

Because of the qualitative character of this study, the purpose was to describe participants’ perceptions as detailed as possible but not to describe how widespread each perception was. Further quantifications were avoided, as the semi-structured nature of the focus group interviews possibly would strongly influence how often a point was made (e.g. when a follow-up question was posed). Points made by a single participant were therefore reported and treated equally important as those made by “some” or “all”.

The questionnaire provided some quantifiable information used in the discussion and conclusion to describe the general tendencies.

Several themes of interest not directly associated with the narrow learning objectives were included in a separate analysis, as they provided valuable insights into escape rooms and debriefings in general; the protocol did not cover this. The study protocol was uploaded to clinicaltrials.gov on 05/03/2021 (ClinicalTrials ID: NCT04783259).

## Results

### Focus group interviews and participants

Eight groups, with a total of 32 participants completed the study. Participants were in their late twenties and evenly distributed amongst gender (see Table 1). In the post-interview questionnaire, they reported prior experience, educational preferences and familiarity (see Table 2). Focus group interview duration had a median of 36 min and ranged from 23 to 43 min. After coding and analysing six focus group interviews, saturation was reached, as no new themes could be identified from the last two focus group interviews. This was confirmed by TJ and OR watching the remaining focus group interviews on video. The remaining two focus group interviews were neither transcribed nor analysed.

**Table 1:** Demography of participants *n* = 32

	Number
Age	
Years (median [IQR])	26 [25–26]
Years (range)	24–32
Sex	
Male	19
Female	13

**Table 2:** Self-reported statistics regarding prior experience, educational preferences and familiarity of participants in the post-interview questionnaire *n* = 32

	Strongly Agree	Agree	Neutral	Disagree	Strongly Disagree
Prior to the study, I had experience with					
Escape rooms	14	12	1	0	5
Escape room-based debriefing	0	1	1	7	23
Medical simulation	18	14	0	0	0
Simulation-based debriefing	9	12	7	2	2
I tend to prefer interactive education as opposed to classroom education	18	14	0	0	0
I am familiar with my fellow participants in the escape room					
On a personal basis	28	3	1	0	0
On a professional basis	7	16	6	2	1

### Themes related to narrow learning objectives

Five main themes were identified from the focus group interviews (Table 2).

1. Within the first theme, *experience with narrow learning objectives*, participants expressed that they did not feel restricted by the narrow learning objectives but experienced the possibility of discussing other topics important to them.
2. *Topics discussed in the debriefing* were mainly about *exchange of information*. Participants understood the term *communication* as broader than *exchange of information*. Participants also discussed *leadership* and *situational awareness*.
3. *Learning mechanisms*: The single and narrow focus was seen to increase the depth and perceived outcome of the debriefing. Participants explained that the debriefer helped maintain focus on the learning objectives and increased the perceived learning outcome by guiding participants in their reflection.
4. *Perceived learning outcomes in relation to exchange of information* that were identified included: Knowledge of different ways of communicating and the importance of optimising communication when working together; skills in ignoring redundant information; and change of attitude by becoming aware that others perceive a situation differently. A few participants reported no learning outcome due to being familiar with the learning objectives prior to the intervention.
5. *Influence of the learning approach* covered the parts of the whole learning experience besides the debriefing, focussing on how each of the different phases affected perceived learning of participants. Participants expressed that the video lecture contained little educational value but supported the rest of the approach by setting the scene. Regarding the scenario briefing, some participants wished for more emphasis on the learning objectives just before entering the room. Some participants explained that they got caught up in the game and did not focus on the learning objectives during the escape room. Finally, participants indicated that they liked the coherence of the experience in that each part supported the next and enabled deeper reflection.

**Table 3:** These focus group interview excerpts were selected to illustrate participants’ experiences and perceived learning outcomes of narrow learning objectives in a medical escape room. Themes are presented in the left column, with main themes in bold. The right column contains focus group interview excerpts translated from Danish to English. Brackets indicate the interviewer and participant in pseudonymised form

**Experience with narrow learning objectives**	
Restrictions	[INT 2, PERS 2] It was a narrow learning objective, but I feel there was freedom to say whatever you wanted.
Wishes for other topics	[INT 5, PERS 3] (…) I think that we discussed it quite well (…) it is not like I feel that there are that many other topics which I need to discuss.
**Topics discussed in debriefing**	
Exchange of information	[INT 2, PERS 1] In some way, I feel that communication is wider. Exchange of information becomes very instrumental in some way, very concrete. Whereas communication is also when you lay a hand on someone’s shoulder to say, “You are doing okay”. I do not necessarily feel that it is an exchange of information. [INT 3, PERS 4] (…) if the question posed was simply “What did you get from it regarding communication?”. If you were presented with that and nothing else, then it would maybe have been things like distribution of roles and short messages, but we would not have come up with all the other stuff with the non-verbal communication and such. (…)
Other topics	[INT 4, PERS 1] (…) I also think that we discussed the other things a lot, but this was in the context of exchange of information.
**Learning mechanisms**	
A single focus	[INT 4, PERS 2] (…) focusing exclusively on the communication improves the communication part, as opposed to focusing on different topics at the same time (…)
Effects of narrow learning objective	[INT 2, PERS 4] (…) it was nice that there was an angle on the discussion we had afterwards, as it makes the outcome greater. (…) we said many things building on something someone else had said earlier, and you would probably not have done so much if all four had had different agendas (…) [INT 6, PERS 1] (…) despite the discussion veering in various directions, you can say ”The purpose of today was investigating communication”.
Keeping focus with facilitation techniques	[INT 4, PERS 3] (…) if we veered off somewhere that was not relevant, we were advised to consider its relevance to communication, and then we got it back on track again. (…)
Facilitation techniques’ effects on learning outcome	[INT 2, PERS 2] (…) I do not at all think that I would have had the same outcome if it (the debriefing ed.) had not been structured. Because verbalising things and getting help perceiving things in our communication, which we had not considered ourselves. If we were just to sit down and talk within the group, then I do not think that we would have gone over it as deeply and become aware of what we had said and in which ways.
**Perceived learning outcomes in relation to exchange of information**	
Knowledge	[INT 2, PERS 2] (…) reflecting on it, I actually used a lot of communication tools, and I have also become aware of ways to communicate (…) [INT 6, PERS 2] It is also a takeaway. (…) thinking about that sometimes you have to do some tasks together. How do we make sure that the communication becomes as good as possible?
Skills	[INT 5, PERS 3] (…) something about learning to sort the redundant away.
Attitude change	[INT 3, PERS 4] (…) that people do not always look at it the same way you do. So, the thing about being concrete in what you say that’s what I took with me the most.
No new learning	[INT 4, PERS 4] We had heard all the stuff before, so I do not know if you learn, but it was like a reminder of how important some of it is. [INT 6, PERS 4] I do not feel that I learned anything new regarding medical expertise.
**Influence of the learning approach**	
Educational value of the video lecture	[INT 3, PERS 4] (…) it was a little, well, trivial. I found my attention wavering at that point. So, I got the most out of it when we discussed it afterwards as it got tied to something concrete. [INT 4, PERS 1] (…) it like set the scene for ”What it actually is we are going to play within this learning session.” [INT 6, PERS 1] (…) if we had not had the lecture, then we might not have been able to remember the stuff there is to communication.
Learning objective in the scenario briefing	[INT 6, PERS 1] (…) if, just before entering when we were briefed about the game, you had mentioned something like ”Remember your communication, remember your closed-loop”, like if you had been reminded about those things. Do you not think that our communication would have been completely different in there?
Learning objective in the escape room	[INT 5, PERS 1] (…) it (the escape room ed.) became more about the challenge, right. So in that way, I did not think much about it (the learning objective ed.) during.
Combination of different learning modalities	[INT 5, PERS 1] (…) at the university then sometimes you just watch a lecture, and then that is what you get out of the teaching in some subject. Where here, it is like a three-pronged approach where you like watch it, then you try it, maybe unknowingly, and then afterwards talk about it and reflect. (…) I think it works very well in terms of learning.

### Other findings

The focus group interviews provided points beyond discussing the narrow learning objectives (Appendix 6). Two main themes were identified. *Meta-learning* regarding the debriefing itself, where participants realised the usefulness of debriefings in an educational context. And *the general experience of the escape room*, where participants stated that the experience was relevant to clinical practice. Some participants also described how learning within an escape room differed from conventional communication training because the lack of formal pressure promoted more genuine communication that reflected real-life behaviour. Furthermore, the format was engaging and fun, and the low requirements on medical expertise were appreciated as they did not steal focus.

### Post-interview questionnaire

The questionnaire results concerning the learning objectives and their perceived learning outcome are presented in Table 3. It shows that all participants experienced learning about exchange of information and many about other topics as well. The vast majority liked the narrow focus of the learning objectives and would not have preferred a broader learning objective.

**Table 4:** Results from post-interview questionnaire *n* = 32

	Strongly Agree	Agree	Neutral	Disagree	Strongly Disagree
In the study learned something about					
Exchange of information	20	12	0	0	0
Other topics	0	15	13	1	3
Liked the narrow focus of the learning objectives	14	12	5	1	0
Would have preferred a broader learning objective	0	2	9	17	4

## Discussion

This qualitative study identified narrow learning objectives and structured debriefing to increase perceived learning depth and general outcome of medical escape room sessions. Using semi-structured debriefing allowed for discussions of other elements relevant to the students.

### Narrow learning objectives were not restricting

Unstructured game-like learning exercises allows for many different learning objectives catering to participants’ interests but can result in superficial and erratic discussions with frequent changes in topics. To increase the learning outcome, there is a need for some structure. According to the questionnaire, most participants preferred a narrow learning objective though they did not have a comparable experience with a broad learning objective. During the focus group interviews, participants did not feel restricted by the narrow learning objectives and felt free to discuss other topics of their interest. This is a benefit of the semi-structured rather than fully-structured debriefing format and illustrates an educational duality: participants feel a need for autonomy but also for being paced by the educator to focus on the learning objectives and return to the topic when getting off-topic. The results suggest that many educators’ fear– that guiding the debriefing is seen as negative by participants [32]– might not have an empirical basis. However, the current setting took several steps to focus on the narrow learning objectives (video lecture, scenario briefing, and debriefing). Therefore, this focus was more stringent than is typical in simulation practice.

### Structuring debriefings affect perceived learning outcomes

By making participants verbalise perceptions and experiences during the escape room and their perception of aspects of the experience related to the narrow learning objective, the educator increased the perceived learning outcome by increasing the depth of the debriefing. Though the research group defines this as facilitation techniques, the participants refer to it as *structure*. This complies with others’ findings that participants prefer structured debriefing sessions [23–25]. This study emphasises that such structure indeed can improve– at least the perceived– learning outcome.

### Medical expertise in the escape room

Participants expressed it as an advantage that the level of ambition for medical expertise in the escape room puzzles was low. If there had been difficult medical challenges, these could have reduced learning related to *exchange of information*. This could be related to matching the amount of new information to avoid an overload, as described in cognitive load theory [33]. It can also be challenging, especially for novice facilitators, not to overwhelm learners, as they might do so to avoid risking the participants perceiving the learning session as boring [34]. This study can make it easier for educators to accept that less can be more: participants see the value of discussing fewer topics in more depth.

### Exchange of information as a learning objective

The learning objectives were “Recognising the different ways of exchanging information” and “Discussing the impact of exchanging information on problem solving”. Participants were thoroughly introduced to the definition of exchanging information and reminded of the learning objectives throughout the learning experience, yet participants widely used the term *communication* during the focus group interview. When asked, participants explained that they perceived *exchange of information* as a more narrow and instrumental term than *communication*. Participants considered the reflections in the debriefing to concern both the instrumental factors, such as structuring a message and taking notes, and elements, such as non-verbal communication and the distribution of roles within the group.

This exemplifies a challenge in concept learning [35]: Educators need to balance conceptual sharpness and keep learners motivated about a new concept. The literature on learning (second) languages shows that it may lead to steeper learning and acceptance curves if skills are presented practically with a focus on implementation instead of insisting on conceptual sharpness in using terms [36–38]. However, this may increase the risk of misunderstanding concepts and terms. Focusing on definitions can be frustrating for many and may slow down learning.

### Limitations

In participant recruitment, the research group may have created a selection bias by having voluntary admissions for the study, thus risking a sample of the general population with a specific interest in innovative and interactive education. This potential bias is of little concern since the aim concerned the learning objectives, not the innovative and interactive education style.

The study design increases the risk of a social-desirability bias. The researchers attempted to pre-empt this by explicitly informing the participants of the importance of enlightening both positive and negative aspects.

As a medical student at UPCH, TJ had met some of the participants before, but none of the interviewers had met the participants. Although it cannot be ruled out that familiarity between participants and TJ affected the debriefing, the data collected during the focus group interview session is without this bias.

## Conclusion

Narrow learning objectives and structured debriefing can increase perceived learning depth of medical escape room sessions. Using semi-structured debriefing still allows for discussions of other elements relevant to the students.

The findings of this study encourage the use of narrow learning objectives and semi-structured debriefings in future conductions of medical escape room sessions. This will hopefully aid educators in choosing suitable learning objectives to optimise the overall learning outcome of medical escape rooms.

### Electronic supplementary material

Below is the link to the electronic supplementary material.

Supplementary Material 1

## Data Availability

Not applicable.

## Abbreviations

CAMES: Copenhagen Academy of Medical Education and Simulation
MERGE: Medical Escape Room Gaming Experience
NTS: Non-technical skills
UCPH: University of Copenhagen
