# Supplementary material for: Perceptions of medical students on narrow learning objectives and structured debriefing in medical escape rooms: a qualitative study
Source: BMC Med Educ. 2024 Apr 11;24:403. doi: 10.1186/s12909-024-05295-4 (PMC11010419; doi:10.1186/s12909-024-05295-4)
Supplement: Supplementary file 1 — Supplementary Material 1 [file 12909_2024_5295_MOESM1_ESM.docx]

**Appendix 1**

| **Questionnaire concerning demographics** |
| --- |
| **First name: Date:**  **Gender: Age in years:** |

Please complete the following questionnaire by placing a CROSS in the appropriate box.

**X**


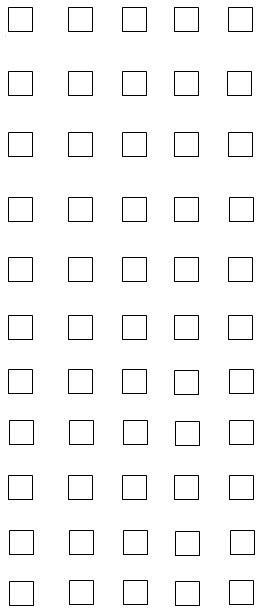


|  | | strongly agree | agree | neutral | disagree | strongly disagree |
| --- | --- | --- | --- | --- | --- | --- |
| 1. | I have experience with escape rooms prior to today’s events |  | | | | |
| 2. | I have experience with escape room based debriefing prior to today’s events |  |  |  |  |  |
| 3. | I have experience with medical simulation prior to today’s events |  |  |  |  |  |
| 4. | I have experience with simulation-based debriefing prior to today’s events |  |  |  |  |  |
| 5. | I tend to prefer interactive education as opposed to classroom education |  |  |  |  |  |
| 6. | I am familiar with my fellow participants in the escape room on a personal basis |  |  |  |  |  |
| 7. | I am familiar with my fellow participants in the escape room on a professional basis |  |  |  |  |  |
| 8. | I learned something about exchange of information today |  |  |  |  |  |
| 9. | I learned something about other subjects today |  |  |  |  |  |
| 10. | I liked the narrow focus of the learning objectives |  |  |  |  |  |
| 11. | I would have preferred broader learning objectives |  |  |  |  |  |

**Appendix 2**

Laparoscopy puzzle

**Equipment list:**

| **Need to have:**   - 2 x laparoscopic forceps - A thin chain - 2 x small flashlights - 1 x plastic box with a lid (70x40cm) - Drape to cover the box - Small table with no top, matching the size of the box - Glue - 6 x pieces of wire - 2-5 x Plastic body parts - Box with key |
| --- |

**Puzzle explained:**

A plastic box stands on a table opposite the manikin. The box is covered in a surgical drape, so only a small hole grants vision to the inside of it. The inside of the box is lit by a flashlight and filled with various fake body parts, such as hands and organs, for effect and as obstacles. A key is visible, dangling from one of the hands and at the other end of the box, a light shines from a hole in the bottom. On the opposite side of the hole in the cover are two holes in the box, allowing to operate two surgical forceps inside the box. In close proximity to the setup hangs the two forceps from the ceiling in the chain, locked to it with a combination lock with the code 2222. The operator will not be able to see inside the box and will have to be verbally guided by a team member looking inside from the opposite side. They will have to secure the key and drop it through the hole in the bottom of the box. The key serves to open another box located underneath the plastic box. Inside this box is a note that says: “*Lock 1 - Nr. 2 = 4”,* and an ALS puzzle for the next puzzle.

**How to setup puzzle:**

1. Supply the plastic box with two holes on one side, sized so the laparoscopic forceps can be operated through them.
2. Fixate the plastic body parts to the floor of the box using a wire.
3. Make a hole of 5cm in diameter in one of the corners opposite of the forceps-holes. Note: Be sure that the hole is reachable with the forceps!
4. Fixate a flashlight to the ceiling of the box with wire.
5. Cover the box with a drape – e.g. surgical drape – glued to the surface so that only a small 20x10 cm hole on the opposite side of the forceps-holes grants vision inside the box.
6. Fixate the box on the topless table with wire in a manner so that it can easily be removed and opened by yourself, but not the participants.
7. Fix the second flashlight with wire to the table underneath the box so that its light shines up on the hole in the corner of the box.
8. Place the locked box containing the next puzzle underneath the plastic box.
9. Hang the laparoscopic forceps with wire from the ceiling close to the plastic box. They have to hang high enough so that they can not reach the box with the forceps before unlocking them.
10. Place the key on one of the body parts, e.g. on a finger, turn on the lights and close the box.

**How participants solve the puzzle and get the number for the combination lock:**

The previous puzzle grants them the code to the laparoscopic forceps. The participants must use these to retrieve the key from inside the box by dropping it through a hole in the floor and verbally guiding each other. This provides them with the key to a box containing the ALS puzzle. Inside the box, there is a note that says: *“Is ALS a puzzle to you? Collect it here on the glass – things might look better from a different side!”* and a puzzle for the next puzzle.

**Laparoscopy**

| “Laparoscopic box” setup | The puzzle in action | | The forceps hanging |
| --- | --- | --- | --- |
| 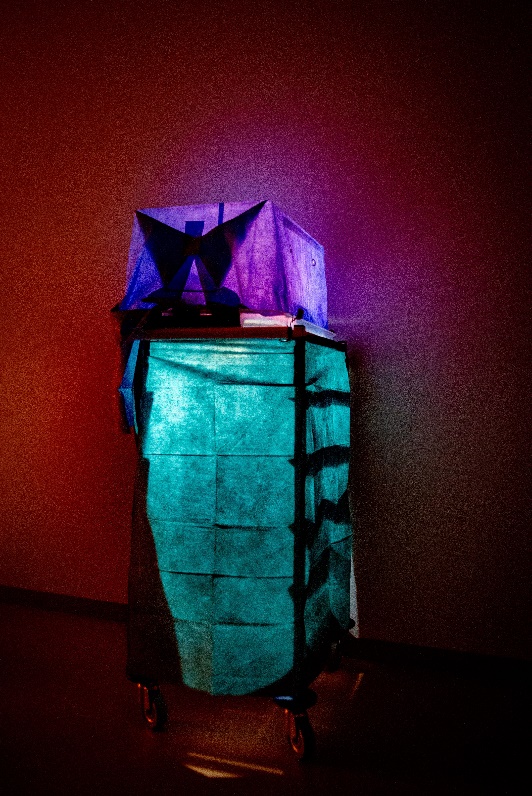 | 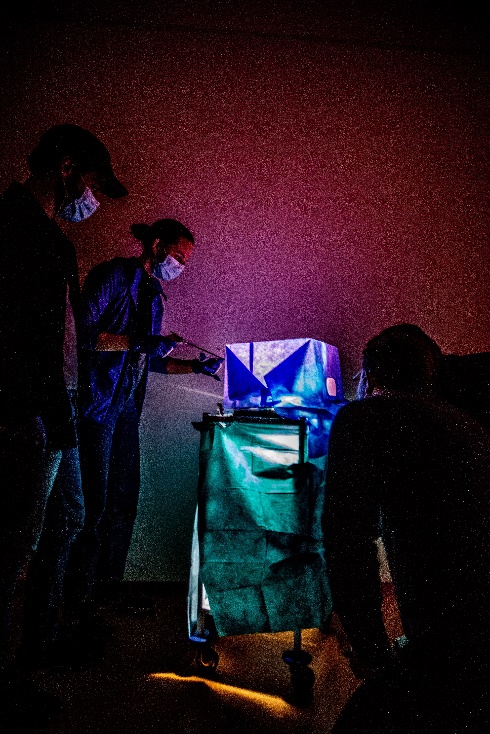 | | 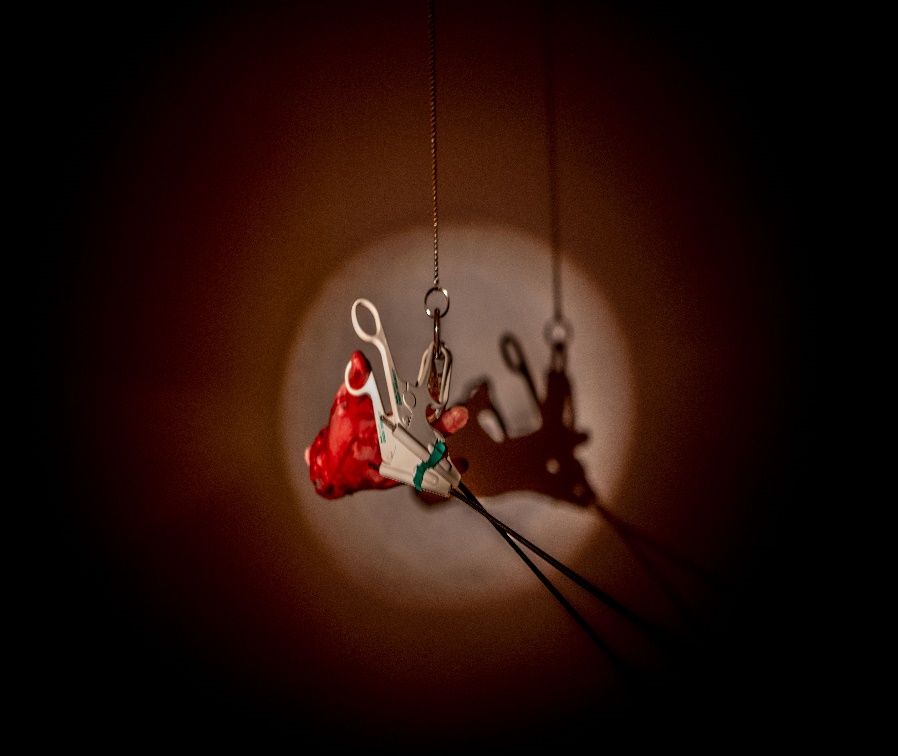 |
| Setup inside the box | | View from outside the box | |
| 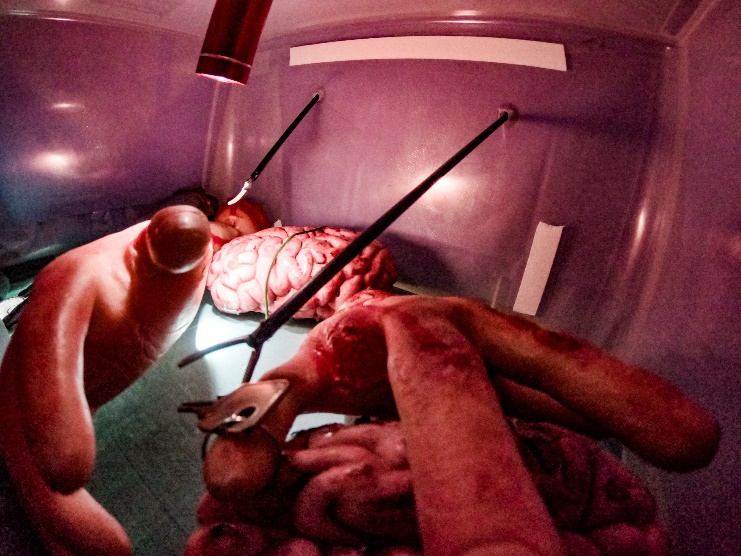 | | 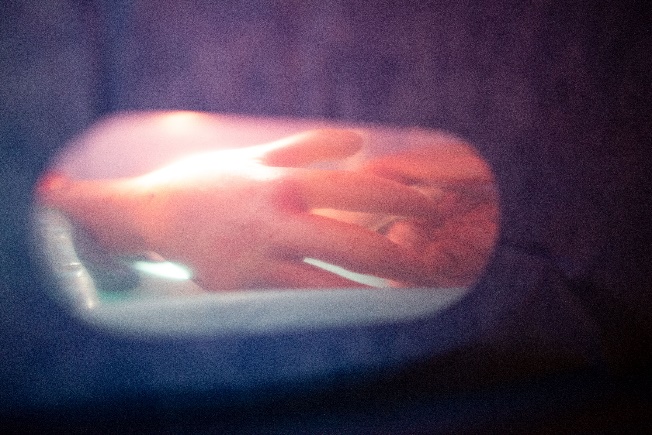 | |

**Appendix 3**

**Link to the video lecture in Danish**

<https://youtu.be/xHSSEAJTHxU>

**Transcript of the video lecture in English**

Hello. My name is Tami, and I am a medical student at the University of Copenhagen and employed at CAMES. I am going to present you with a video lecture concerning the exchange of information. In this lecture, I will start by talking about the purpose of discussing this topic. I am going to address the content of information exchange, I will address the channels used to exchange information, I will address the challenges in information exchange, I am going to talk about the barriers in information exchange, and I will talk about the tools you can use when you are to exchange information.
 When we talk about exchange of information here at CAMES, we have to define which skills we are talking about. We consider technical skills and cognitive and social skills. Technical skills are skills like inserting an IV, using an algorithm such as ABCDE, using software, giving a diagnosis and many other practical medical skills. Cognitive and social skills, on the other hand, are skills like situational awareness and decision-making, which are some of the cognitive skills used, and leadership, teamwork and problem-solving, which are social skills related to interaction. As a subtheme to teamwork, we locate exchange of information. It is this subtheme we are going to be concerned about today.
 Let me give you some examples of why exchange of information might be relevant for us as physicians. An example of when we are exchanging information as physicians could be in the transfer of a patient to another physician at the end of a shift. Here, we look at an exchange of information from one person to another. It is rarely as simple that exchange of information is one-way, though. Often, it will be a two-way communication, where the other person asks questions, and there is exchange both ways. The situation might be even more complicated, e.g., when you have to pass information to multiple participants, such as a nurse and a physician from another department as well. It might even be that multiple persons have to pass information about this patient to multiple persons, who also ask questions back; if you have a nurse and an attending doctor who also need to pass on information, it becomes a larger and more complex exchange of information. You also experience situations in clinical practice where you have a trauma and a lot of different parties communicate among themselves and between each other. Then, information exchange becomes a larger and more complex concept than what you initially think. Therefore, this is important to learn about.
 First, I will focus on the content of an information exchange. When we address a simple information exchange between two people, we have some content. We have a topic that the information exchange concerns, and then the relation between the two participants who have influence on the content of the information exchange. If you are familiar with each other, it affects the content one way, whereas if your relationship is professional, it affects the content another way. So, we both have the topic discussed and the relation between the participants, which together constitute the content of an information exchange.
 I will also define the channels used to exchange information. Overall, there are two channels: The verbal channels, i.e. what we say and how we say it, whether we say a lot or a little, and whether we are very concrete or very descriptive, and whether we have a demeaning tone or are constructive in our way of speaking. In addition, there are all the non-verbal elements used in our communication and exchange of information. It is elements such as our body language, our facial expressions and our positioning in relation to each other.
 Some of the possible challenges in exchange of information could, e.g. be disruption of the channel used in the exchange. An example of this is something as basic as conferring a patient over the phone when one of the phones runs out of battery, and the channel is cut off. Another example could be noise; you could be sitting in an ambulance where the sirens make it difficult to hear each other. Or a room might be very dark, making it difficult to see each other. It is all the different disruptions that worsen the practical elements of the exchange. In addition, the amount of information exchanged can result in a problem in the capacity of the recipient in that it can be difficult to differentiate the relevant parts of the information. As an example, if you provide a lot of different points whereas only one is important, the important point might be lost. In relation to this information overload, it might also be that the recipient is exposed to many other stimuli, such as people talking to them, which also imposes challenges on the recipient’s capacity. There are also mental challenges in exchange of information. I would like to do a little exercise with you. I want you all to think of a house. I suppose that you are all thinking of a house now? Here you see some different pictures of houses. Possibly, you were thinking of one of these houses; maybe it was a completely different house, but all of you probably did not think of the same house. This is an example of how a word can have different meanings to different recipients.
 Now, we are going to talk about different barriers to the exchange of information. Barriers can be in the relationship between two participants. If we have a medical student and an attending physician from a different department, the hierarchy might impose a barrier to the medical student, who might not dare provide all the details as they fear challenging the other participants’ authority. There are also different aspects, but here I just provide one example of each barrier. We also see barriers concerning the content, i.e. the topic or the relation that I mentioned earlier. Here, I will provide an example of the topic. It could be that the topic in the information exchange is taboo, so it might be awkward, or the participants might have problems addressing the topic and withholding information. We also see personal barriers in the exchange of information. This might be that you are a new physician in a field and thus insecure about your own clinical skills. Then you might withhold information, as you are insecure whether they are correct. In addition, there might be barriers related to the culture in the department. If the department is busy and people do not spend much time transferring the patients to each other, then the exchange of information will be of lower quality.
 Last, I will round off by addressing tools in exchange of information. We have some practical, technical tools such as the ISBAR algorithm and closed loop. These tools can be utilised for structure and quality assurance; I am not going to explain them in-depth here. In addition, this lecture should have taught you that the recipient has to be focused when listening to the information, as there are many challenges and barriers in exchange of information. Knowing these challenges and barriers can contribute to awareness about them, making it possible to try and work around them. Last, you have to be ready to throw away the technical tools if you feel that they are a hindrance. You should not use them just because you know them, you should use them because they aid you in exchange of information.
 To sum up the lecture. The purpose of the lecture is to show that the exchange of information is very common in clinical practice. It can be difficult, and it is very important to be good at it. The content in an information exchange both concerns the topic and the relation of the participants. An exchange of information has one or more channels, both verbal and non-verbal. Challenges are plenty, both concerning the challenge, the amount of information and the recipient’s capacity. Barriers are plenty as well. They can concern the relation, personal barriers, the content, etc. The tools are knowing about all these parts of information exchange, which are important to know about. If you know them, then you are better prepared to work with them and avoid making mistakes caused by the challenges. Thank you for your attention.

**Appendix 4**

| **Phase** | **The manuscript for the educator** (Examples of formulated sentences) |
| --- | --- |
| 1. *Setting the scene (2 min)* | - “In this debriefing, we are going to reflect on your experiences. It will take about 45 minutes. We will first discuss shortly, what happened in the escape room. Then we are all going to reflect on how you shared information. Lastly, we will look at what you can take with you.” |
| 1. *Reaction  (8 min)* | - “How are each of you feeling right now?” - “Are you sitting with any technical questions about the puzzles or the escape room in general at this moment?“ - “Now then, having answered your questions, I am going to continue with the debriefing.” |
| 1. *Description  (10 min)* | Recognising the different ways of exchanging information   - “We are – in this debriefing – especially interested in how you shared information with each other. We could discuss many things, but we would like to focus on this aspect.” - “I want each of you to answer the question: ‘What were the different ways you used to exchange information?’ I want you to describe how you did it in as much detail as possible.” *Start with one and then ask the others for additions. Consider taking notes that all can see.* - *If you noticed any additional examples of information exchange, lead their attention to it and make them describe it by saying something like:* “I also noticed a point where … can you elaborate what happened there?” |
| 1. *Analysis  (15 min)* | Discussing the impact of exchanging information on problem-solving   - “What went well in today’s game in regards to the exchange of information, and how did it affect your performance in the escape room?” - “How and why would the progression have been different if you had not been allowed to talk to each other?” - “Were there phases of the escape room where the exchange of information was more or less important? Please elaborate.” - “Did you experience any situations where you found it difficult to exchange information with a member of your team? When and why?” - “How could you have optimised your exchange of information?” |
| 1. *Application (10 min)* | - “How can the things you learned today be applied in your clinical practice?” - “Lastly, I want each of you to mention the most important thing that you learned from today’s events.” |

**Appendix 5**

| **Subject** | **Questions** |
| --- | --- |
| *Interview instruction* | - Welcoming and introduction. - Explain the background of the study and emphasize the volunteer character. - Emphasize that we are interested in “honest” opinion, also points that bothered or were perceived as challenging. - Emphasize that there is no right or wrong and that we are interested in participants’ views. |
| *The escape room* | - “How was your experience with the escape room? Is there anything you want to emphasise?” |
| *The debriefing* | - “What do you think about the debriefing?” - “What are the pros and cons of a structured debriefing like you had versus a different format, e.g. an unstructured conversation?” - “Would you have preferred that the format had been less structured? Why/why not?” - “How do you feel about the very narrow focus of the debriefing, i.e. the exchange of information?” - “Were there any topics that you wanted to discuss but could not due to the narrow learning objective?” - “Would you rather have had more time to discuss certain aspects? - After your consideration in this interview, I ask again: what do you think about the debriefing? |
| *The learning outcome* | - “When during the escape room and/or debriefing do you feel that you learned something concerning exchange of information?” - “Did you learn anything besides exchange of information? What and when?” - “Have you experienced that the debriefing has affected your learning outcome today? How and why?” |

**Appendix 6**

| These focus group interview excerpts were selected to illustrate interesting utterances of participants, not directly concerning the study’s aim. Themes are presented in the left column, with main themes in bold. The right column contains focus group interview excerpts translated from Danish to English. Brackets indicate the interviewer and participant in pseudonymised form. | |
| --- | --- |
| **Meta-learning** | [INT 2, PERS 1] I also think I learned something about what debriefing can do. In my head, a debriefing is something you do after something serious has happened (…) But it can also be utilised in many different situations, just to agree on how to improve something or “what did you experience?”. So, it is a very good tool. |
| **The general experience of the escape room** |  |
| Relevance for clinical practice | [INT 2, PERS 2] (…) You could find different scenarios and see yourself in a clinical setting and think, “Gee, I can actually use this” when you come back to clinical practice. |
| Comparison to conventional communication training | [INT 2, PERS 1] (…) Because that focus splits a bit from communication, which is very much in the forefront in the normal settings, then it also becomes much more real as it is in everyday clinical practice, where you do not necessarily think much about your communication. (…)  [INT 2, PERS 1] Yes, it can feel a bit artificial in the (conventional ed.) scenario training sometimes. I feel that I assume a role that I do not necessarily have in a clinical setting (…) It is, of course, a version of how I am in a clinical setting, but it is not one to one.  [INT 2, PERS 4] (…) Normally, we are assigned to random teams with unfamiliar people (…) It was fun (in the escape room ed.) being able to (…) experience how you communicate, when you are not nervous, and there is not anything at stake.  [INT 6, PERS 3] (…) in normal simulation training, it is always like: “now we have done as it said on the list. The doll is alive”. |
| Engagement and learning value | [INT 3, PERS 1] (…) it would be positive if it had been a learning session and the focus had still been on zombies (…) it makes it a bit more like a game. It becomes like, “now we have to learn this,” but it becomes, like, presented differently. In some way, it also makes it a bit more fun to participate in. |
| Low requirements for medical expertise | [INT 4, PERS 2] Mostly because you then were able to focus on that, the focus was information exchange, and we did not pause in information just because there were medical technical things we could not remember. |
